# Characterization of Vaccine Tweets During the Early Stage of the COVID-19 Outbreak in the United States: Topic Modeling Analysis

**DOI:** 10.2196/25636

**Published:** 2021-09-14

**Authors:** Li Crystal Jiang, Tsz Hang Chu, Mengru Sun

**Affiliations:** 1 Department of Media and Communication City University of Hong Kong Hong Kong Hong Kong; 2 College of Media and International Culture Zhejiang University Hangzhou China

**Keywords:** topic modeling, social media, infoveillance, vaccine, coronavirus, COVID-19

## Abstract

**Background:**

During the early stages of the COVID-19 pandemic, developing safe and effective coronavirus vaccines was considered critical to arresting the spread of the disease. News and social media discussions have extensively covered the issue of coronavirus vaccines, with a mixture of vaccine advocacies, concerns, and oppositions.

**Objective:**

This study aimed to uncover the emerging themes in Twitter users’ perceptions and attitudes toward vaccines during the early stages of the COVID-19 outbreak.

**Methods:**

This study employed topic modeling to analyze tweets related to coronavirus vaccines at the start of the COVID-19 outbreak in the United States (February 21 to March 20, 2020). We created a predefined query (eg, “COVID” AND “vaccine”) to extract the tweet text and metadata (number of followers of the Twitter account and engagement metrics based on likes, comments, and retweeting) from the Meltwater database. After preprocessing the data, we tested Latent Dirichlet Allocation models to identify topics associated with these tweets. The model specifying 20 topics provided the best overall coherence, and each topic was interpreted based on its top associated terms.

**Results:**

In total, we analyzed 100,209 tweets containing keywords related to coronavirus and vaccines. The 20 topics were further collapsed based on shared similarities, thereby generating 7 major themes. Our analysis characterized 26.3% (26,234/100,209) of the tweets as *News Related to Coronavirus and Vaccine Development*, 25.4% (25,425/100,209) as *General Discussion and Seeking of Information on Coronavirus*, 12.9% (12,882/100,209) as *Financial Concerns*, 12.7% (12,696/100,209) as *Venting Negative Emotions*, 9.9% (9908/100,209) as *Prayers and Calls for Positivity*, 8.1% (8155/100,209) as *Efficacy of Vaccine and Treatment*, and 4.9% (4909/100,209) as *Conspiracies about Coronavirus and Its Vaccines*. Different themes demonstrated some changes over time, mostly in close association with news or events related to vaccine developments. Twitter users who discussed conspiracy theories, the efficacy of vaccines and treatments, and financial concerns had more followers than those focused on other vaccine themes. The engagement level—the extent to which a tweet being retweeted, quoted, liked, or replied by other users—was similar among different themes, but tweets venting negative emotions yielded the lowest engagement.

**Conclusions:**

This study enriches our understanding of public concerns over new vaccines or vaccine development at early stages of the outbreak, bearing implications for influencing vaccine attitudes and guiding public health efforts to cope with infectious disease outbreaks in the future. This study concluded that public concerns centered on general policy issues related to coronavirus vaccines and that the discussions were considerably mixed with political views when vaccines were not made available. Only a small proportion of tweets focused on conspiracy theories, but these tweets demonstrated high engagement levels and were often contributed by Twitter users with more influence.

## Introduction

### Background

The COVID-19 pandemic has affected more than 200 countries and territories, killed more than 1.2 million people, devastated the global economy, and disrupted the daily life of billions of people [1]. Owing to the lack of effective containment measures during the early stages of the COVID-19 outbreak, many of those heavily affected placed their hope on the development of coronavirus vaccines. Ever since the early stages of the outbreak, extensive news coverage followed the progress of vaccine developments, while web users engaged in heated discussions about coronavirus vaccines or vaccines in general on various social media platforms such as Facebook, Twitter, and Instagram [2-4]. It is crucial to understand media portrayals and public discussions of coronavirus vaccines during the early stages of the outbreak because they influenced policy-making in public health and public perceptions of and attitudes toward vaccination in the later stage [5-11]. A comprehensive understanding of the public opinion during the initial phase of infectious outbreaks will inform how public health professionals and policymakers make decisions in addressing public concerns in future outbreaks of infectious diseases [12].

### Infodemic and Early Stages of Outbreaks

Frequent infectious outbreaks are an ongoing reality for globalized societies, and the early stage of an outbreak is always challenging. The beginning of an outbreak is typically characterized by a lack of accuracy, widespread misinformation, as well as heightened uncertainty and fear among the general public [13,14]. In the first couple of months of the COVID-19 pandemic, policymakers had limited knowledge about coronavirus and largely relied on data modeling for predictions and decisions. Similarly, owing to the lack of knowledge, there was little consensus among media professionals, public health professionals, and politicians over containment measures [15]. Instead, geopolitical discourses, conspiracy theories, and racial bigotry created significant amounts of noise for officials trying to manage the pandemic [16-19]. All of these issues brought intensified fear and anxiety to the public.

Social media platforms shape public experience and opinions, while also serving as platforms for public health. During the initial phase of the pandemic, social media became the hotspot of all sorts of issues for the pandemic. Previous studies have shown that social media content about COVID-19 is mixed with a deluge of stigmas, rumors, and misinformation [16-18] and is highly biased by political and social ideologies [19-21]. On February 15, 2020, the World Health Organization officially coined a phenomenon “infodemic,” which refers to the rapid spread of misinformation through social media platforms and other outlets on a global scale [22-25]. An infodemic is a serious threat to public health as it greatly advocates hostile attitudes toward preventive measures and complicates our fight with the COVID-19 pandemic [26].

### COVID-19, Vaccines, and Social Media

Despite the scientific consensus that vaccination is a safe and effective approach to prevent infectious diseases, there is more controversy over the use of vaccines than over other preventive measures (eg, hand hygiene, social distancing). These concerns include fear of side effects, uncertainty about vaccine efficacy, and general mistrust of the sciences and the government. These contentions have resulted in vaccine hesitancy, declines in immunization, and even small outbreaks of vaccine-preventable diseases [27-29]. Controversies over vaccination have often manifested in social media communities, leading to increasing research investigating the spread of information and opinions about vaccines on various social media platforms. This inquiry mainly focuses on the intensified competition between provaccination and antivaccination views on social media in recent years [30]. Both manual coding and computational methods have identified similar proportions of provaccination and antivaccination content on YouTube and Twitter [31-33], but antivaccination content—produced by closely connected communities and employing sophisticated antivaccination advocacy strategies—often outweigh the provaccination content [34,35]. There is some variation across specific types of vaccines. For example, influenza-related videos contain more anti-immunization content compared to videos on measles, presumably because influenza vaccination is normally perceived as new and less efficacious [32].

Scholars propose several strategies for tackling the vaccine controversy and addressing antivaccination information on social media [23,24], such as infoveillance. Infoveillance is an emerging approach that tracks what people do and write on the internet to reflect public opinions, behaviors, knowledge, and attitudes related to health issues [36]. Major applications of infoveillance include but are not limited to monitoring health-relevant messages on the internet (eg, antivaccination sites), outlining web-based health information availability (eg, vaccine advocacies), and analyzing search engine queries to predict disease outbreaks (eg, syndromic inquiry). By analyzing social media posts related to public health issues, previous studies have successfully performed surveillance on public opinions and public sentiments [36-40], predicted prevalence and mortality across time and space [41,42], and explained how intended or unintended behavioral responses are shaped by social networks and other information features [43-45]. In the case of analyzing vaccine-related social media messages, infoveillance can provide key stakeholders (eg, health organizations, governments) the benefits of revealing public concerns over vaccines and monitoring public sentiments in real-time. It also helps identify influencers and advocates, directly engages with the vaccine targets (ie, people who are at high risk of infection), and manage misinformation and hostile messages efficiently. Infoveillance is particularly powered by big data and computational techniques as they offer very useful tools for understanding social media content in an unstructured, bottom-up manner. Previous studies have successfully used computational methods to examine public perceptions on influenza vaccine [46], human papilloma virus vaccines [47], and childhood vaccinations [48,49].

This study aims to investigate the discussion related to coronavirus vaccines on Twitter during the early stage of the COVID-19 outbreak in the United States (February 20, 2020 to March 31, 2020). This study will contribute to our understanding of coronavirus vaccines and connections to attitudes related to vaccines by tracking back to the initial public concerns. The findings of this study will elucidate the public discussions on new vaccines or vaccines under development, and the concerns and issues revealed in this study can show the implications on public health efforts in coping with infectious disease outbreaks in the future. Provided that the coronavirus vaccines show plenty of uncertainty in efficacy and effectiveness, using unsupervised learning methods, we aim to explore the main themes that emerged from the tweets related to coronavirus vaccines during the initial stage of the pandemic in the United States (RQ1). We also seek to examine how these themes evolved over time (RQ2).

Out of the different types of misinformation, conspiracy theories have merged as a significant concern in the “social media infodemic.” Since the COVID-19 pandemic, several studies have analyzed certain types of conspiracy theories such as the coronavirus as a bioweapon [49], the 5G coronavirus [50], or “Film Your Hospital” [51]. However, few studies have looked at the overall spread of conspiracy theories related to COVID-19. A recent analysis of German tweets indicated that less than 1% of the tweets analyzed were related to conspiracy theories, although partisanship boosted the spread of conspiracy theory tweets [52]. This study examined how conspiracy theories related to coronavirus vaccines were represented in the American tweets at the early stage of the outbreak (RQ3).

Previous studies also indicate that the spread of conspiracy theories and antivaccination messages follow a different pattern compared to that of provaccination messages. On social media, antivaccination content, in general, attracts more likes and engages more discussion because content producers are inclined to use a variety of persuasive strategies (eg, health narratives) and present antivaccination in the form of public criticism aggressively [30,53]. Antivaccination messages are normally produced by a small proportion of powerful influencers, but antivaccination supporters perpetuate echo chambers by actively spreading conspiracy theories and misinformation through a more decentralized network [34]. This study also expected some differences in the influences (ie, number of followers) and engagement levels when comparing different themes in Twitter vaccine discussions. Specifically, compared to the tweets discussing other vaccine-related themes, tweets discussing conspiracy theories were likely contributed by Twitter users with more followers (H1a) and produced more engagement than tweets that discuss other themes (H1b).

## Methods

### Data Source

The study period was set from February 20 to March 31, 2020. We marked this period as the early stage because it corresponded to a sharp increase in the coronavirus case count and death toll in the United States (eg, over 181,000 cases and 3606 deaths by March 31, 2020). At the end of March 2020, the United States became the country with the most number of confirmed cases in the world. Moreover, in March 2020, most state and local governments declared COVID-19 as a public health emergency, issued stay-at-home orders, and mandated closures of schools and public meeting places [54]. We purposely chose this time frame to capture the tweets during the first phase of the COVID-19 outbreak in the United States. Meltwater [55], a commercial web-based media monitoring service, was used for data collection. Meltwater has access to the full Twitter pipelining data hosting service, providing customized reporting options with the last 15 months of Twitter history. Meltwater geotagged each tweet using the user’s Twitter bio-related or other geo-related information, thus ensuring that all tweets included in the sample were posted by American Twitter users.

Using the social media monitoring and data collection platform provided by Meltwater, we collected tweets originating from the United States and written in English that were related to the coronavirus vaccine by using the following Boolean query: (covid OR coronavirus) AND (vaccine OR vaccines OR vaccination OR vaccinations OR vaccinate OR vax OR vaxine OR vaxx OR vaccinated). Using this strategy, we identified 117,718 tweets (including original tweets and quote tweets but not replies and retweets). The text of the tweet and relevant metadata, including username, date of the post, and follower count, were stored. We also stored the engagement metric provided by Meltwater, which was a composite score representing how many times a tweet was retweeted, quoted, liked, or prompted a reply by other users. A higher engagement value indicated that the tweet received more attention by other Twitter users.

### Topic Modeling

To analyze the obtained data set, we applied topic modeling—an unsupervised machine learning algorithm that allows researchers to uncover hidden thematic structures in a sizable collection of documents [56]. A topic model can “produce a set of interpretable topics (groups of words that are associated under a single theme) and assess the strength with which each document exhibits those topics” [57]. In this study, we used Latent Dirichlet Allocation (LDA), one of the widely used topic models that groups words that frequently co-occur in documents into various topics. By providing the text input and setting the desired number of topics, LDA automatically produces a set of topics, words are allocated to the topics, and the topic proportions are attributed for each document [58]. We decided to use LDA, as findings yielded by prior studies indicate that it performs well with both long and short texts. In addition, it has been previously used to examine COVID-19–related discussions on Twitter [59].

### Data Preprocessing

To prepare the corpus for LDA topic modeling, we first removed the quoted content within the quote tweets and the “QT” (meaning a quote tweet) to retain only the original content of the tweet. As the length of document plays a significant role in the topic modeling method [60], tweets with fewer than 5 words were removed, leaving a total sample of 100,209 tweets. Following this, all the URLs within the tweets were removed. Next, the tweets were preprocessed using standard natural language processing practice [61]. We converted all the letters to lower case, removed all the stop words (eg, the, it, that), lemmatized the words, and removed numbers, white space, emoticons, symbols, and punctuation, with the use of Python packages such as NLTK (Apache) [62] and spaCy (Explosion AI) [63]. Bigram and trigram were also created and added. After tokenization, Document-Term-Matrix was built and used for the LDA topic modeling.

### Number of Topics

**Figure 1:**
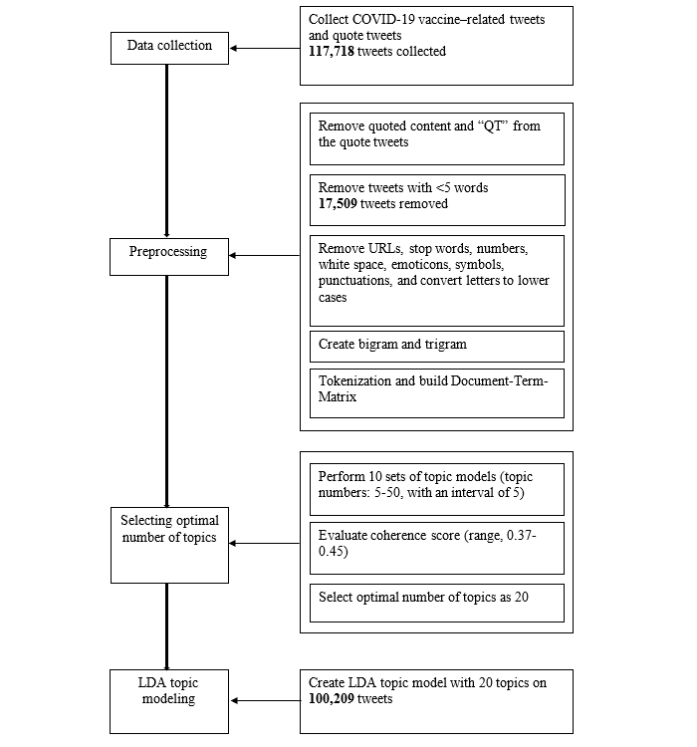
Data processing and analysis flowchart. LDA: Latent Dirichlet Allocation; QT: quote tweet.

To determine the optimal number of topics for this tweet set, we performed 10 sets of topic models with topic numbers ranging from 5 to 50 (with intervals of 5) by implementing the LDA model from the Python package MALLET. The topic coherence —a metric focusing on the interpretability—of the 10 topic models were then calculated and evaluated for selecting the appropriate number of topics [64,65]. We decided to use the topic model with 20 topics in this study because it presented the highest topic coherence as compared with the other candidate models. Figure 1 presents the steps of data processing and creating topic models.

### Topic Interpretation and Further Analyses

The output of the LDA topic model based on 20 topics was reviewed. Although the LDA model presumed that each document contained a mixture of topics and the model produced a probability topic distribution for each topic, we considered only the dominant topic, that is, the topic with the highest probability in that document, and categorized each tweet subject to its dominant topic [66,67]. We then reviewed the 20 top-associated terms, together with the top 5 tweets with the highest topic percentage contribution of each topic, before labeling each topic. These labels were based on the authors’ background knowledge regarding vaccine hesitancy as well as the observation of coronavirus vaccine–related news and user-generated opinions on Twitter during the data collection and analysis [68]. The 3 authors involved in this study independently labeled the topics, and the resulting 3 sets of topic labels were compared. The diverse topic labels were discussed and 100% agreement between the authors was reached. The labeled topics were further grouped into distinct themes deductively following discussion. Lastly, differences between the themes on the number of followers and levels of engagement were examined. Nonparametric tests were used, as the outcome variables were not normally distributed within the current data.

## Results

### Topic Modeling

We analyzed 100,209 tweets in this study. The average number of followers of each tweet was 19,300.62 (SD 431,794.41), and the average engagement value of the tweets was 29.41 (SD 624.91). To examine themes that have emerged in coronavirus vaccine discourses on social media (RQ1), LDA modeling with 20 topics was performed. During the labeling process, it was noticed that 4 of the topics were related to news concerning human trials and testing of coronavirus vaccines. As the 4 topics were similar and closely related, the 3 authors agreed to merge these discussions into 1 overall topic, that is, *News of Vaccine Development*. Next, the remaining 17 topics were organized into 7 themes. The themes, topic labels, and associated words for each topic are presented in Table 1. The majority of the tweets were labeled as *News Related to the Coronavirus and Vaccine Development* (26,234/100,209, 26.2%) and *General Discussion and Seeking of Information on the Coronavirus* (25,425/100,209, 25.4%), followed by *Financial Concerns* (12,882/100,209, 12.9%), *Venting Negative Emotions* (12,696/100,209, 12.7%), *Prayers and Calls for Positivity* (9908/100,209, 9.9%), *Efficacy of Vaccines and Treatments* (8155/100,209, 8.1%), and *Conspiracies about Coronavirus and Its Vaccines* (4909/100,209, 4.9%).

**Table 1:** Themes and topics from coronavirus vaccine discussions on Twitter.

Themes/topics of discussion				Associated words		Frequency of discussion (N=100,209)		Percentage of discussion		Examples of tweets			
**News related to coronavirus and vaccine developments**													
	News of vaccine developments			human, trial, begin, volunteer, receive		17,435		17.4%		As said by the US authorities, the first clinical trial of COVID-19 vaccine on humans has been planned to begin today. The first human subject is going to get the dose today.			
	News of US government research funding/plans for the pandemic			research, fund, system, medical, government		4012		4.0%		The White House approved the emergency fund to deal with COVID-19 in the United States and abroad. The fund will support the development of the COVID-19 vaccine by providing money for new equipment as well as supplies.			
	News of research plans for vaccines			pandemic, develop, effort, outbreak, step		4787		4.8%		COVID-19: Mainland China has taken a new step for developing the vaccine. A team from around the world will investigate the initial results on youngsters.			
**General discussion and seeking of information on coronavirus**													
	Seeking of information on vaccines			question, understand, information, real, cure		3460		3.5%		I would like to know how the coronavirus vaccine interacts with the flu shot. Although I am not that clever to tell if we should be concerned about it, I want to raise this question out of curiosity.			
		Discussion about coronavirus trend		case, number, low, current, increase		3268		3.3%		This will never work. For example, there is a rising number of confirmed cases in the Republic of Korea and Taiwan after the ease of restrictions. There is going to be nonstop waves of infection if there is no vaccine. The main purpose of isolation is reducing the load on the health care system.			
		Discussion about coronavirus and its vaccines		virus, spread, vaccine, fast, mutate		4860		4.8%		Although the mutation of coronavirus is much slower than that of the flu viruses, it is an RNA virus, which normally mutates nearly 100 times faster than viruses based on DNA. It will be much difficult to control or vaccinate in the future if millions of people are infected by it as it will provide more chance for the coronavirus to mutate.			
		Comparisons with influenza		flu, kill, deadly season, thousand		10,145		10.1%		First, we have the flu vaccine already. Second, compared with the influenza and the Spanish flu that have caused over 50 million deaths, the coronavirus seems more infectious. Third, compared with that with the Spanish flu, the death rate with the coronavirus is higher. Fourth, while the influenza virus has more impact on individuals older than 65 years, the coronavirus does not discriminate individuals according to age.			
		Preventive measures		protect, hand, safe, force, home		3692		3.7%		The following steps can help in defeating COVID-19: stay calm and keep washing your hands with water and soap or use hand sanitizer. Keep social distancing, open doors with your elbow, and do not rub your nose, face, or shake hands with others.			
**Financial concerns**													
		Disparity in income		American, rich, poor, capitalism, afford		5653		5.6%		We, the taxpayers, are going to pay for the research on COVID-19 vaccines, which we deliver to the select few without any compensation. Rich people can acquire billions from tax cuts and chief executive officers can acquire millions from compensation. The capitalism of the Republican Party is socialism for the rich. We all are the targets.			
		Price of vaccine		free, affordable, cost, charge, insurance		7229		7.2%		The COVID-19 vaccine should be free of charge for people who do not have enough money for copayment for insurance or those who do not have medical insurance. The fee of my vaccination will be covered by my insurance, and I am able to pay for the difference. We have to ensure that the health insurance companies pay their part first.			
**Efficacy of vaccines and treatments**													
		Efficacy of vaccines		prevent, cancer, infect, immunity, antibody		4096		4.1%		A lot of people don’t know about the COVID-19 vaccines. They are not injecting your body with the dead virus but harmless spikes. Immunity will be built to the spikes after injecting the vaccine. This could ease the worries of those opposing the vaccine.			
		Efficacy of treatments/preventions		test, treatment, effective, hospital, prove		4059		4.1%		Lately, many physicians from the United States and France have asserted the effectiveness of antimalarial medication in treating COVID-19. Does it mean that the malaria vaccine would work against COVID-19 also?			
**Conspiracies about coronavirus and its vaccines**													
		Conspiracies related to companies/stock/government		profit, market, stock, government, attempt		4909		4.9%		Is it possible that the Republican Party and Trump manipulated the stock market and profited through insider trading of Moderna’s stock? This biotechnology company, which invented the new vaccine, had its stock increased by 15%.			
**Venting negative emotions**													
		Negative emotions (toward Trump and big pharmacies)		wrong, damn, business, stupid, idiot		7019		7.0%		The vaccine makers could create whatever they want. Even if someone got injured or died, we cannot sue them. If someone dies, that’s just bad luck. If some child dies, that’s just bad luck. If someone becomes paralyzed, that’s just bad luck. The profits of the pharmacies grow because we never fight back. We are just the slaves of the big pharmacies.			
			Trump-related		trump, lie, truth, blame, reality		5677		5.7%		Agreed. What Trump and his incompetent administration do is to lie about everything: the seriousness of the disease, keeping the disease on a tight rein already, getting a vaccine soon.		
**Prayers and calls for positivity**													
			Emotions/prayers		good, hope, happen, pretty, remember		5228		5.2%		That is some good news! All of us need some optimism.		
			Calls for positivity		great, love, call, idea, good		4680		4.7%		I enjoy seeing the positivity in the current state.		

### Themes and Topics From Coronavirus Vaccine Discussions on Twitter

#### News Related to the Coronavirus and Vaccine Development

During the COVID-19 pandemic, social media users frequently shared news related to coronavirus as well as the development of coronavirus vaccines. There were 3 topics under this theme: *News of Vaccine Development*, *News of US government Research Funding/Plans for the Pandemic*, and *News of Research Plans for Vaccines*. Tweets categorized in this theme included general news on the progress of human trials and vaccine development across different countries (eg, Germany), announcements of US government funding for scientists and companies conducting research, and upcoming prevention plans for the pandemic released by official bodies as well as coronavirus vaccine research across the globe (eg, “The White House has permitted an emergency funding of US $1 billion overall in order to fight the COVID-19 outbreak. The emergency fund will offer resources as well as financial support for COVID-19 vaccine development for the states”).

#### General Discussion and Seeking of Information on the Coronavirus

A total of 5 topics were grouped under this theme: *Seeking of Information on Vaccines, Discussion of the Coronavirus and Its Vaccines, Discussion of Coronavirus Spread and Infection Trends, Comparisons with Influenza, and Preventive Measures*. The coronavirus was often compared with the influenza virus in terms of death rate, speed of transmission, and so on (eg, “Up till now, there is no cure for COVID-19 but only treatment for the symptoms. The long-term plan is to invent a new vaccine; yet, there would be no vaccine available in the next couple of months”). The importance of preventive measures, including handwashing and social distancing, was also stressed because there is currently no vaccine nor effective treatment for the coronavirus infection (eg, “We all need to get rid of bad habits. Stop touching your face when you are in public space. Scratch your nose only after washing your hands or scratch it with your sleeve. And remember to wash your hands once you get home”).

#### Financial Concerns

There were 2 topics under this theme: *Disparity over Income* and *Price of the Vaccines.* In the topic *Disparity over Income*, conversations were related to the gap between the rich and the poor during the pandemic as well as the differences in access to future coronavirus vaccines (eg, “All this is turning into a class war now. Only rich people can get the COVID-19 vaccine as none of us can be sure that the vaccine will be affordable for everyone”). Worries of inequality brought about by capitalism in obtaining vaccination were also expressed (eg, “Capitalism should never get closed to health care systems. The operating costs of the traditional Medicare and the administrative costs of the US health spending is extremely high. The new vaccine should be free for everyone”). As for the price of vaccination, “free” instead of “affordable” coronavirus vaccines for all Americans were urged (eg, “Citizens who were not able to pay for the COVID-19 vaccine will just keep spreading the disease. The COVID-19 vaccine should be affordable or free for everyone!”).

#### Venting Negative Emotions

This theme had 2 topics: *Negative Emotions (toward Trump and big pharmaceutical companies)* and *Trump-related frustrations.* Negative emotions, including anger and disappointment, toward Donald Trump or big pharmaceutical companies, were presented, as those Twitter users believed that Trump and Big Pharma were trying to profit from the pandemic. Additionally, negative emotions were expressed toward Trump explicitly owing to claims he made that are believed to have been mistaken, such as the claim that receiving the influenza vaccine would prevent COVID-19 (eg, “He [Trump] actually believed that a flu shot could fight COVID-19. I do not understand how people with brains elected this guy”).

#### Prayers and Calls for Positivity

The 2 topics under this theme were *Emotional Expressions/Prayers* and *Calls for Positivity*. Tweets allocated within these 2 topics included messages that aimed to encourage others during the pandemic, expressed hopes for and needs for effective coronavirus vaccines, and hopes for an end to the pandemic (eg, “Let us hope that the COVID-19 situation will be resolved when we have a vaccine/cure for it!”).

#### Efficacy of the Vaccine and Treatment

The 2 topics under this theme were *Efficacy of Vaccine* and *Efficacy of Treatment/Prevention*. These topics stressed the uncertainties of how well the vaccines for coronavirus work as well as the effectiveness of the current treatment and prevention strategies (eg, “I have learnt from some journals that medicines such as chloroquine, hydroxychloroquine, and azithromycin could be used as treatment or prophylaxis of COVID-19. I hope such treatments can help buying us time while getting a vaccine for COVID-19”).

#### Conspiracies About Coronavirus and Its Vaccines

There were different conspiracies about coronavirus and its vaccines on social media (RQ3). Many of these were related to the companies developing coronavirus vaccines, stock markets, as well as the government. For example, some tweets were claiming that the coronavirus vaccine would contain a microchip that would allow the government or company to track the vaccine receivers (eg, “Once the COVID-19 vaccines are launched, people will be motived by fear to receive the vaccines that have microchips in it.”). There were also claims that the US government spread the coronavirus deliberately and withheld the coronavirus vaccines (eg, “I think the US has the cure already because it invented this bioweapon. It does not want other parties to wreck its cautiously crafted plans for devastation and racketeering”)

### Themes Across Time

**Figure 2:**
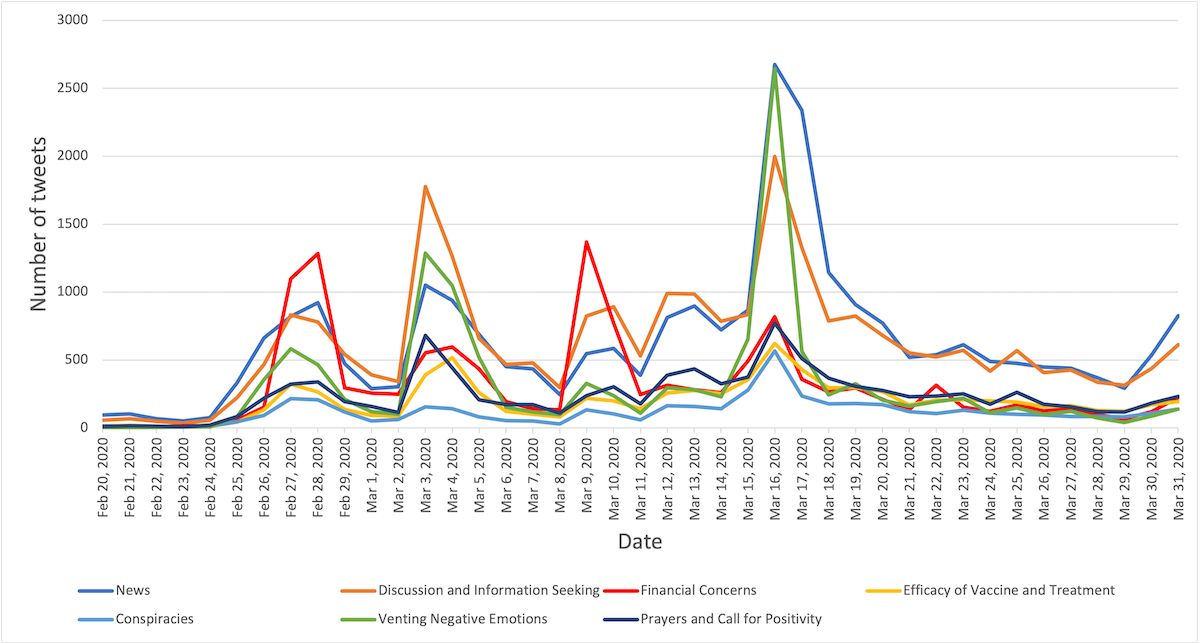
Frequencies of themes of the tweets over time (February 20, 2020 to March 31, 2020).

Figure 2 shows the changes in the coronavirus vaccine–related discussions on Twitter based on the themes identified across the data collection period, that is, February 20, 2020 to March 31, 2020 (RQ2). Among the 7 themes identified, *News Related to Coronavirus and Vaccine Development* and *General Discussion and Seeking of Information on Coronavirus* were the most frequently presented overall. Coronavirus vaccine–related discourses on Twitter were promoted by breaking news or announcements and speeches made by the governments and political elites. As shown in Figure 2, there were several peaks in the coronavirus vaccine–related discussions at the early stage of the outbreak. The discussions were elevated on March 16, 2020, which corresponded to the trending news that the Trump administration was attempting to offer large sums of money to a German company in exchange for exclusive access to a possible coronavirus vaccine on March 15, 2020. Coronavirus vaccine discourses regarding *Financial Concerns* reached the peak and exceeded other themes on February 28, 2020 and March 9, 2020. The rising discussions about the prices and affordability of the coronavirus vaccines were related to Health and Human Services Secretary Alex Azar’s refusal of promising affordable coronavirus vaccines for all US citizens on February 27, 2020 and Bernie Sanders’ promises of free coronavirus vaccine for all Americans on March 9, 2020, respectively. We observed some co-occurring patterns across the themes during the same peaks and time periods. General discussions and information on coronavirus highly mirrored the themes of news related to coronavirus and vaccine developments. The expression of negative emotions also increased when the discussions of these 2 themes reached a spike. There were also some observed differences in the themes across time. The efficacy of the vaccine and treatment, conspiracies about the coronavirus and vaccines, and prayers and calls for positivity appeared more periodically, while other themes (ie, news related to coronavirus and vaccine developments, general discussion and information on coronavirus, financial concerns, venting negative emotions) were more episodic, featured with several peaks instigated by breaking news or events related to vaccine developments.

### Differences in the Follower Numbers and Engagement Level

#### Analyses of Follower Numbers and Engagement Level

H1a and H1b hypothesized that conspiracy tweets’ contributors had more followers, and conspiracy tweets received higher levels of engagement than tweets with other themes. To examine the differences between themes in the number of followers and levels of engagement, further analyses were performed. First, the results of classification of LDA topic modeling were attached in the original data (which contained the metadata, including the number of followers and engagement metric of each tweet). The data were then entered into the SPSS software (IBM Corp). Next, we created a categorical variable according to the themes and used it as the independent variable to examine the differences in the number of followers and levels of engagement across themes by using the Kruskal-Wallis *H* test. Post-hoc analysis was also performed using the Bonferroni-corrected Dunn test. As the hypotheses focus on the difference between conspiracy tweets and the other tweets with themes that presented attitudes and concerns toward the coronavirus vaccines, tweets labeled as news or discussion/information seeking were excluded from the analyses. Table 2 presents the median and mean rank of numbers of followers and levels of engagement.

**Table 2:** Median and mean ranks of the followers and engagement among themes.

Themes	Followers		Engagement	
	Median	Mean rank	Median	Mean rank
Financial concerns	685	24,411.87^a^	1	20,836.52^a^
Efficacy of vaccines and treatments	740	24,957.68^a,b^	1	20,784.34^a^
Conspiracies about coronavirus and vaccines	770	25,095.54^b^	1	20,631.67^a^
Venting negative emotions	616	23,615.43^c^	0	19,275.31^b^
Prayers/calls for positivity	620	23,776.11^c^	1	20,807.51^a^

^a-c^Same superscripts in the same column indicate no significant statistical differences (*P*>.05); different superscripts in the same column indicate significant statistical differences (*P*<.05).

#### Followers

The results of our study suggested that there were significant differences in the number of followers between different themes (*χ^2^_4_*=77.8, *P*<.001). The post-hoc test further suggested that conspiracy tweets were more likely to be posted by users with a large number of followers than the tweets classified as *Venting Negative Emotions* (*P*<.001), *Prayers and Calls for Positivity* (*P*<.001), and *Financial Concerns* (*P*=.04). Tweets that discussed the efficacy of vaccines and treatments were also more likely to be posted by users with a large number of followers than the tweets classified as *Venting Negative Emotions* (*P*<.001) and *Prayers and Call for Positivity* (*P*<.001). Similarly, tweets expressing financial concerns were more likely to be posted by users with a large number of followers than the tweets classified as *Venting Negative Emotions* (*P*<.001) and *Prayers and Calls for Positivity* (*P*=.007). As conspiracy tweets were more likely to be posted by users with more followers than the tweets identified as *Financial Concerns, Venting Negative Emotions,* and *Prayers and Calls for Positivity*, H1a was partially supported.

#### Engagement Levels

The results of our study suggested that there were significant differences in the levels of engagement between different themes (*χ^2^_4_*=155.8, *P*<.001). The post-hoc test further suggested that tweets classified as *Venting Negative Emotions* significantly received lower levels of engagement than tweets classified as *Conspiracies about Coronavirus and Its Vaccines* (*P*<.001), *Efficacy of Vaccines and Treatments* (*P*<.001), *Prayers and Calls for Positivity* (*P*<.001), and *Financial Concerns* (*P*<.001). As conspiracy tweets only received higher levels of engagement than the tweets classified as *Venting Negative Emotions*, H1b was partially supported.

## Discussion

### Principal Findings

This study examined how American Twitter users discussed coronavirus vaccines during the initial stage of the COVID-19 pandemic. Using the technique of topic modeling, this study identified 7 themes in Twitter discussions. While approximately one-fourth of the tweets were about news updates related to coronavirus and vaccine developments, the remaining tweets consisted of general discussion and information seeking on coronavirus, expressions of financial concerns, disclosures of negative emotions, prayers and calling for positivity, discussions of vaccine and treatment efficacy, and conspiracy theories. In a close association with news or events related to vaccine developments, some themes demonstrated episodic changes and high degrees of co-occurrences. However, the themes of conspiracies about coronavirus and vaccines, prayers and calling for positivity, and efficacy of vaccines and treatments appeared in more periodic patterns. This study enriches our understanding of the public concerns related to vaccines during the early stage of the outbreak, and these shared concerns can inform public health organizations and professionals for more tailored health messages and vaccination policies.

Our results suggest that during the early stage of the pandemic, Twitter discussions related to coronavirus vaccines were centered on general policy issues and were largely mixed with political discussions. Two contextual factors presumably contributed to such characteristics. First, because key stakeholders did not quickly achieve a consensus on containment measures in the initial phase of the pandemic, vaccines were often staged in the public discourses as a potential remedy [12]. It is also understandable that when there was no specific vaccine available, individuals and communities addressed the vaccine issues from a policy-related perspective by discussing the investment and cost aspects of vaccination. Second, the discussions on coronavirus vaccines were situated in the political discourses during the presidential election. A topic revealed from this study was negative emotions toward Donald Trump and explicitly for his claim of using influenza shots to prevent coronavirus infections. Other COVID-19 studies also similarly demonstrated that Donald Trump and other politicians deeply influenced the vaccine discussions and even contributed to the spread of misinformation [34]. This was not surprising as vaccination is one of the politicized health controversies [52,69,70], and it was a strategic effort to feature the vaccine in political discourses. However, political disagreement over vaccines could be detrimental because they were often associated with vaccine hesitancy, reduced confidence in scientific and health facts [71], and decreased policy support for immunizations [72]. Recent research suggested that as different vaccines passed phase trials and were made available to the public, the discussions over vaccine efficacy and safety sharply increased in the United States [73].

Consistent with other studies that examined coronavirus vaccine sentiments and attitudes on social media over different periods of the pandemic [34,74,75], our study indicates that the public had mixed opinions and emotions over coronavirus vaccines, which may create significant barriers to reaching the vaccine-induced herd immunity. Antivaccination arguments and conspiracy theories were one of the major sources for vaccination opposition, although they did not constitute a large part of social media discussions. However, this small proportion of tweets was contributed by Twitter users with more influence. They also demonstrated higher engagement levels, thus resulting in echo chamber effects among small-size subnetworks [52]. It is observed that most themes demonstrated peaks and troughs over time but some themes (eg, *Conspiracies* and *Efficacy of Vaccines and Treatments*) were more periodic and some themes (eg, *Venting Negative Emotions*) were more episodic. We speculate that conspiracies and efficacy concerns were largely about unconfirmed but expected issues (eg, pharmacy conspiracies apply for all the vaccines); thus, such discussions were likely to merge periodically. However, unexpected events (eg, Trump’s claim of using influenza shots to prevent coronavirus) will stimulate heated discussions, leading to a peak in the data. When these events were later addressed by the authority’s responses, the discussions gradually vanished.

### Practical Implications

This study offers several practical implications for addressing the infodemic at the early stage of outbreaks or health crises. First, public health professionals should timely and appropriately address the public needs for vaccine-related information. Our analysis revealed that many Twitter discussions were by people seeking more information or expressing concerns on coronavirus and vaccines. Such surges in information demand should be addressed by supplying with appropriate information that is easy to follow.

Second, health communication may differentiate communication strategies for episodic and periodic themes. As indicated by the results, episodic themes (eg, financial concerns, venting negative emotions) tended to emerge when breaking news or unexpected events occurred. Quick and appropriate responses to these events would effectively reassure the public and eliminate “epidemics of fear” [76]. For periodic themes such as conspiracy theories and efficacy concerns, regular surveillance and tailored responses can counterbalance the negative effects of these themes.

Health organizations and health professionals should make more systematic and organized efforts to address antivaccination content and other vaccine-related misinformation. Together with other studies [47-49], this study indicated that antivaccination content and misinformation about vaccines were contributed by closely connected communities and followed several clear and predictable patterns. When coronavirus vaccines were still under development, antivaccination content had been spreading on the internet along with these recurring conspiracy themes, which indicates that the battle with conspiracies and antivaccination messages is a long fight. A prebunking approach could effectively reduce the negative outcomes of conspiracy theories and misinformation about vaccines [77]. For example, recent research shows that attitudinal inoculation (eg, prewarning the audiences with common vaccine-related conspiracy theories) can develop resistance to the influence of vaccine conspiracy theories at a later stage [78].

Last but not the least, social media influencers (ie, accounts with many followers) play an important role in the spread of vaccine-related opinions. Fact-checking the content published by social media influencers may effectively limit the spread of conspiracy theories, which requires efforts from both social media platforms and the influencers themselves [79,80]. Twitter recently made some initial moves by introducing a labeling and striking system to identify and remove COVID-19 misinformation [81]. In a related vein, social media influencers are also encouraged by social media and health organizations to enhance their health literacy and their capacities for fact-checking before they self-proclaim as vaccine activists or public health activists on social media platforms.

### Limitations

This study has the following limitations. First, the study findings are limited to the Twitter discussions during the first phase of the COVID-19 pandemic in the United States. The public’s concerns might have changed over time as the development of vaccines progressed. The recent suspension of a coronavirus vaccine owing to adverse effects has brought a lot of discussions on vaccine safety [9]. The peculiar political environment (eg, presidential election year) may also have contributed to the patterns of the results. Further research is encouraged to look at discussions related to coronavirus vaccines on different social media forms and in different countries. Longitudinal studies and comparisons across countries or regions are particularly preferred to examine the dynamics and heterogeneity in the spread of information and opinions. This study only captured the Twitter discussions during the first stage of the COVID-19 pandemic. Future research could employ larger data sets from Twitter or other social media platforms, especially the latest data sets, to reveal the bigger picture of public concerns over coronavirus vaccines.

There are also some limitations in this analysis. For example, we relied on keyword inquiry to extract vaccine tweets from a database, but we cannot guarantee that all posts were related to coronavirus vaccine conversations. Some outliers might have been included in the data. When interpreting the topic themes, although the 3 authors independently coded the 20 topics, the intercoder reliability was not calculated owing to the small number of topics revealed from the LDA results. The analysis also did not distinguish the nature of Twitter accounts, which may be a mixture of personal, organizational, and bot accounts. Bot accounts may have contributed to a certain portion of the Twitter discussions, but we did not estimate the potential bot traffic. Because Twitter data did not account for users’ demographics, while we had a limited understanding of the types of users engaged in the discussion (ie, the number of followers), we do not know more details about the users who were contributing to the discourse. Provided the difficulty of manual classification, future studies should seek to apply more sophisticated machine learning techniques to identify the types of Twitter accounts—ideally, the characteristics of personal accounts (eg, political ideology). Such knowledge will allow us to go beyond the aggregated data to look at individual users.

### Conclusion

Overall, the spread of information and opinions on social media platforms during the early stage of the outbreak has profoundly affected individuals’ beliefs and attitudes toward vaccines and, ultimately, their vaccination decisions. During the early stage of the COVID-19 pandemic in the United States, Twitter discussions related to coronavirus vaccines were centered on general policy issues and were largely mixed with political discussions. The public discussions demonstrated mixed concerns for coronavirus vaccines even before the vaccines were available, and some concerns appeared periodically. These issues call for more preparatory work to cope with the infodemic challenge and to handle infectious breaks in the future.

## Abbreviations

LDA: Latent Dirichlet Allocation
